# Vitamin D did not reduce multiple sclerosis disease activity after a clinically isolated syndrome

**DOI:** 10.1093/brain/awad409

**Published:** 2023-12-12

**Authors:** Helmut Butzkueven, Anne-Louise Ponsonby, Mark S Stein, Robyn M Lucas, Deborah Mason, Simon Broadley, Trevor Kilpatrick, Jeannette Lechner-Scott, Michael Barnett, William Carroll, Peter Mitchell, Todd A Hardy, Richard Macdonell, Pamela McCombe, Andrew Lee, Tomas Kalincik, Anneke van der Walt, Chris Lynch, David Abernethy, Ernest Willoughby, Frederik Barkhof, David MacManus, Michael Clarke, Julie Andrew, Julia Morahan, Chao Zhu, Keith Dear, Bruce V Taylor, the PREVANZ Investigators, Val Gebski, Thomas Kimber, Alan Barber, Paul Wraight, Sandeep Sampangi, Rashida Ali, David Miller, Lauren Krupp, Leonid Churilov, Michael Ching, Susanne Hodkinson, Ernie Butler, Cameron Shaw, Claire Fraser, John Mottershead

**Affiliations:** Department of Neuroscience, Central Clinical School, Monash University, Melbourne, VIC 3004, Australia; Florey Institute of Neuroscience and Mental Health, University of Melbourne, Melbourne, VIC 3010, Australia; Department of Diabetes and Endocrinology, The Royal Melbourne Hospital, Parkville, VIC 3010, Australia; National Centre for Epidemiology and Public Health, Australian National University, Canberra, ACT 0200, Australia; Department of Neurology, Christchurch Hospital, Christchurch 8011, New Zealand; Department of Neurology, School of Medicine and Dentistry, Griffith University, Southport, QLD 4222, Australia; Florey Institute of Neuroscience and Mental Health, University of Melbourne, Melbourne, VIC 3010, Australia; Department of Neurology, John Hunter Hospital, Newcastle, NSW 2305, Australia; Brain and Mind Research Institute University of Sydney, Sydney, NSW 2050, Australia; Department of Neurology, Sir Charles Gairdner Hospital and Centre for Neuromuscular and Neurological Disorders and Perron Institute, University of Western Australia, WA 6009, Australia; Department of Radiology, Royal Melbourne Hospital, Melbourne, VIC 3010, Australia; Department of Neurology, Concord Hospital, University of Sydney, Sydney, NSW 2139, Australia; Department of Neurology, Austin Health, Melbourne, VIC 3084, Australia; Faculty of Medicine, Dentistry and Health Sciences, The University of Melbourne, Melbourne, VIC 3010, Australia; Florey Institute of Neuroscience and Mental Health, University of Melbourne, Melbourne, VIC 3010Australia; University of Queensland, Centre for Clinical Research, Brisbane, QLD 4029, Australia; Department of Neurology, Flinders University College of Medicine and Public Health, Adelaide, SA 5042, Australia; Neuroimmunology Centre, Department of Neurology, Royal Melbourne Hospital, Melbourne, VIC 3010, Australia; CORe, Department of Medicine, University of Melbourne, Melbourne, VIC 3010, Australia; Department of Neuroscience, Central Clinical School, Monash University, Melbourne, VIC 3004, Australia; Midland Neurology, Hamilton, Waikato 3240, New Zealand; Department of Neurology, Wellington Hospital, Wellington 6021, New Zealand; Department of Neurology, Auckland Hospital, Auckland 1023, New Zealand; Department of Radiology and Nuclear Medicine, Amsterdam UMC, Vrije Universiteit, Amsterdam 1081 HV, The Netherlands; Queen Square Institute of Neurology and Centre for Medical Image Computing, University College London, WC1N 3BG, UK; University College London Queen Square Institute of Neurology, Queen Square MS Centre, London WC1N 3BG, UK; Metabolomics Australia (WA), School of Biomedical Sciences, University of Western Australia, Perth, WA 6009, Australia; Neurosciences Trials Australia, North Melbourne, VIC 3051, Australia; Multiple Sclerosis Australia, North Sydney, NSW 2059, Australia; Department of Neuroscience, Central Clinical School, Monash University, Melbourne, VIC 3004, Australia; Department of Statistics, School of Public Health, University of Adelaide, SA 5005, Australia; MS Research Flagship, Menzies Institute for Medical Research, University of Tasmania, Hobart, Tasmania 7000, Australia

**Keywords:** multiple sclerosis, clinical trial, vitamin D

## Abstract

Low serum levels of 25-hydroxyvitamin D [25(OH)D] and low sunlight exposure are known risk factors for the development of multiple sclerosis. Add-on vitamin D supplementation trials in established multiple sclerosis have been inconclusive. The effects of vitamin D supplementation to prevent multiple sclerosis is unknown.

We aimed to test the hypothesis that oral vitamin D_3_ supplementation in high-risk clinically isolated syndrome (abnormal MRI, at least three T_2_ brain and/or spinal cord lesions), delays time to conversion to definite multiple sclerosis, that the therapeutic effect is dose-dependent, and that all doses are safe and well tolerated.

We conducted a double-blind trial in Australia and New Zealand. Eligible participants were randomized 1:1:1:1 to placebo, 1000, 5000 or 10 000 international units (IU) of oral vitamin D_3_ daily within each study centre (*n* = 23) and followed for up to 48 weeks. Between 2013 and 2021, we enrolled 204 participants. Brain MRI scans were performed at baseline, 24 and 48 weeks.

The main study outcome was conversion to clinically definite multiple sclerosis based on the 2010 McDonald criteria defined as either a clinical relapse or new brain MRI T_2_ lesion development.

We included 199 cases in the intention-to-treat analysis based on assigned dose. Of these, 116 converted to multiple sclerosis by 48 weeks (58%). Compared to placebo, the hazard ratios (95% confidence interval) for conversion were 1000 IU 0.87 (0.50, 1.50); 5000 IU 1.37 (0.82, 2.29); and 10 000 IU 1.28 (0.76, 2.14). In an adjusted model including age, sex, latitude, study centre and baseline symptom number, clinically isolated syndrome onset site, presence of infratentorial lesions and use of steroids, the hazard ratios (versus placebo) were 1000 IU 0.80 (0.45, 1.44); 5000 IU 1.36 (0.78, 2.38); and 10 000 IU 1.07 (0.62, 1.85). Vitamin D_3_ supplementation was safe and well tolerated.

We did not demonstrate reduction in multiple sclerosis disease activity by vitamin D_3_ supplementation after a high-risk clinically isolated syndrome.

## Teaser

Low serum vitamin D levels are a known risk factor for the development of multiple sclerosis. However, in a randomized controlled trial, Butzkueven *et al.* found that daily oral vitamin D_3_ supplements did not delay the conversion of high-risk clinically isolated syndrome to clinically definite multiple sclerosis.

## Introduction

Multiple sclerosis (MS) is an inflammatory and neurodegenerative disorder of the CNS that can result in significant accrual of disability over time.^1,2^

MS aetiology is complex,^2^ with multiple known genetic, environmental and infectious risk factors.^3-6^ Conspicuous amongst these are low vitamin D status as measured by low serum levels of 25-hydroxyvitamin D [25(OH)D]^7^ and/or low exposure to sunlight.^7,8^ These are thought to contribute significantly to the robust latitudinal gradient of MS incidence and prevalence^9,10^ and associations between low 25(OH)D levels/low sun exposure levels and MS risk have been reported from multiple observational epidemiological studies,^7,11-14^ including from nested case-control studies,^14^ where blood samples were taken well before MS onset, a meta-analysis showing low gestational vitamin D status is associated with higher offspring MS risk,^15^ and a study showing that lower neonatal 25(OH)D levels are associated with higher MS risk in adolescence and adulthood.^16^ Collectively these studies make reverse causality an unlikely explanation of the association. Furthermore, Mendelian randomization studies consistently show that genetically determined higher serum 25(OH)D levels are associated with reduced risk of MS.^17^ Evidence also indicates that exposure to low sunlight/low vitamin D is important throughout the life course from conception to at least the clinical onset of MS.^8,18^

Vitamin D and sun exposure are linked. In Australia and New Zealand (and most other locations), most of an individual's vitaminD derives from endogenous synthesis following exposure of the skin to solar ultraviolet (UV) radiation. Absorption of UVB photons by 7-dehydrocholesterol in epidermal cells causes conversion to pre-vitamin D_3_ and then vitamin D_3_, which is taken into the circulation. In the liver, vitamin D_3_ is hydroxylated to 25(OH)D_3_, and this metabolite is in turn hydroxylated to a more active form [1,25(OH)_2_D_3_], principally in the kidney but also in many other tissues including immune cells.^19^ Dietary and supplemental vitamin D (in the form of vitamin D_2_, ergocalciferol or vitamin D_3_, cholecalciferol) are additional sources of vitamin D.

In view of the epidemiological links between low 25(OH)D levels, or low sun exposure, and MS risk, there have been several clinical trials of vitamin D supplementation in established MS.^20-22^ Observed effects have been modest at best and largely on non-clinical parameters (e.g. MRI, immune cell function studies). Indeed, three recent meta-analyses concluded that, overall, there was little effect of oral vitamin D supplementation on MS disease activity and in some instances, higher doses of oral vitamin D were potentially associated with increased disease activity.^23-25^ Nearly all the published trials were conducted in relapsing-remitting MS using an add-on design to established interferon-β immunomodulatory therapy. They were of relatively small size and consequently underpowered to detect a moderate therapeutic effect of vitamin D supplementation. The only prior monotherapy study in clinically isolated syndrome (CIS)^26^ was a small pilot study conducted at a single centre in severely vitamin D deficient subjects; this found a significant benefit of vitamin D supplementation on relapse and MRI disease activity.

A monotherapy trial design in people with CIS should markedly improve power as the rate of new inflammatory activity (MRI T_2_ lesions, clinical relapses) can be expected to be higher than that observed in an add-on design to effective therapy. Therefore, trials enrolling participants with CIS at high risk of recurrent events and therefore conversion to MS provide a more powerful and ethically alternative trial design to assess any potential therapeutic effect.^27,28^ Of the documented risk factors for progression to definite MS (as defined by the 2010 McDonald criteria)^28,29^ after CIS, the strongest is the presence of MS-typical MRI lesions.^30,31^

We conducted the PREVANZ study to determine if oral vitamin D_3_ (cholecalciferol) supplementation was superior to placebo in reducing conversion to clinically definite MS (CDMS) either radiologically or clinically, after a CIS with an abnormal MRI. The hypotheses to be tested were that: (i) treatment with vitamin D_3_ supplementation reduces the risk of clinical and MRI progression to MS; (ii) there is a dose-response effect with increasing doses of vitamin D_3_ having a larger treatment effect; and (iii) treatment with vitamin D_3_ at all doses is safe and well tolerated.

## Materials and methods

This phase IIb randomized double-blind placebo-controlled trial compared daily oral doses of vitamin D_3_ [either 1000, 5000, or 10 000 international units (IU)] to placebo in adult CIS cases with a high risk of conversion to MS. The trial was conducted in 23 academic MS centres across Australia and New Zealand commencing April 2013 and finishing with the last visit in December 2020.

### Ethics and oversight

Ethics approval was obtained from the Human Research Ethics Committees of all participating sites. All participants signed written informed consent. The trial was monitored for compliance with Good Clinical Practice standards by an external contract research organization (Neuroscience Trials Australia) and was conducted in accordance with the Declaration of Helsinki. Safety oversight was provided by an independent safety committee. The trial was prospectively registered with the Australian Clinical Trials Registry ACTR12612001160820.

### Randomization and masking

Randomization was at the study centre level using a block randomization method developed externally to the study (Supplementary material, ‘Section 1’). All participants received identical capsules containing their allocated treatment with full double-blinding maintained throughout.

### Eligibility

Inclusion criteria were age 18–65 years, with a new onset of first ever clinical episode of CNS demyelination (optic neuritis, transverse myelitis, brainstem syndrome, other) as determined by a study neurologist, and an abnormal MRI with at least three CNS T_2_ lesions suggestive of demyelination meeting the Paty criteria A or B (lesions must be ovoid and >3 mm).^32^ During the study, with the evolution of MS diagnostic criteria,^29^ we amended the eligibility criteria to allow one lesion in either the spinal cord or the optic nerve to count as one of the minimum three CNS lesions. Participants agreed to refrain from use of MS disease-modifying therapy (DMT), non-trial supplemental vitamin D and sunbed exposure during the trial period. Exclusion criteria included progressive onset MS, other causes of CNS demyelination/inflammation [including neuromyelitis optica spectrum disorders (NMOSD), myelin oligodendrocyte glycoprotein associated disease (MOGAD) and acute disseminated encephalomyelitis (ADEM)], contraindication or unwillingness to undergo MRI, unwillingness to forgo vitamin D or DMT, history of hypercalcaemia or hyperparathyroidism, a history of renal calculi or hyperuricaemia, an estimated glomerular filtration rate < 60 ml/min, a history of any condition requiring treatment with vitamin D or calcium, intercurrent pregnancy or breast feeding. For full inclusion and exclusion criteria, see Supplementary material, ‘Section 2’.

### Study procedures

#### Screening

After referral to the study, potential participants underwent a screening visit to determine eligibility as per the inclusion/exclusion criteria. If deemed eligible, a study MRI was organized [with images then forwarded to the central reading site at University College London (UCL) to confirm eligibility], and screening blood tests were performed. If all eligibility criteria were met, a baseline visit was organized within 2 weeks.

#### Baseline visit

Randomization was undertaken based on the randomization schedule (Supplementary material, ‘Section 1’), and study medication was dispensed. Subsequently the study protocol for visits and testing (Supplementary material, ‘Section 3’) was followed.

#### Follow-up

Monthly phone contact and 12-weekly in-person follow-up visits up to 48 weeks. At each study visit (by phone or in person) a comprehensive assessment of symptoms suggestive of a clinical relapse, adverse events (AEs), new medications, and the development of other health problems was undertaken.

#### Clinical relapse

A clinical relapse was defined based on the 2010 McDonald criteria^28^ as any new or worsening neurological symptom that lasted for >24 h and is associated with a worsening of the Expanded Disability Status Score (EDSS) by at least 1 point, and/or an increase in one of the following KFS (Kurtzke Functional Scores) of at least 1 point, pyramidal, visual, sensory, brainstem or cerebellar. Isolated increases in bowel/bladder or cognitive/mental KFS scores did not constitute a relapse. New or worsening neurological symptoms were not considered a relapse if they occurred in the context of infection or fever or were considered a pseudo-relapse by the treating neurologist. All relapses were confirmed as true relapses by the treating neurologist.

#### Radiology

MRI data were from the baseline and 24- and 48-weeks standardized brain MRIs. All brain MRIs were read at UCL and included rapid notification of conversion to CDMS radiologically at 24 and 48 weeks (full methodology in Supplementary material, ‘Section 4’).

#### Biochemistry

Blood samples were collected at screening, baseline and at each 12-weekly visit. Serum was extracted and stored in 1 ml aliquots at −80°C in a central laboratory. All samples were assayed for 25(OH)D_3_ concentration at the end of the study at The University of Western Australia (UWA) using a liquid chromatography tandem mass spectrometry assay standardized under the international Vitamin D Standardisation Program (Supplementary material, ‘Section 5’). Notably, a baseline 25(OH)D level was not measured as part of the inclusion/exclusion criteria.

#### Other data collected at baseline

Included self-reported skin colour (with reference to standard photographs, categorized as fair, medium-fair, medium olive, olive, dark), smoking (‘Are you a regular smoker now?’), and body mass index (BMI). All participants had an EDSS recorded by an EDSS certified neurologist. In addition, we recorded the latitude of each study centre.

#### Vitamin D_3_ supplement preparation and quality control

Identical capsules containing vitamin D_3_ or placebo were prepared by LIPA Pharmaceuticals. During the study, six batches of study medication were provided. Independent analysis of vitamin D_3_ content was undertaken by Chemical Analyses Australia Pty Ltd at 3, 6, 12, 18 and 24-month time points; a variation of 10% from the specified content was permitted. The first batch was overlapped with the second batch and testing found that the concentration of vitamin D_3_ as measured decreased after 18 months. No participant received study medication older than 18 months. Thus, for all subsequent batches an 18-month expiry date was used; no significant variations in vitamin D_3_ concentrations were found from the marked dosages. All supplement supplies were delivered to a central pharmacy in temperature-controlled transport with monitoring (with no significant violation of temperature controls), and participants were asked to store the trial supplement at <25°C.

#### Power calculations

With the aim of detecting heterogeneity among the four arms in the rate of conversion to CDMS, power calculations were undertaken through simulation. Simulations assumed 10% dropouts, 30% longer conversion-free survival in the upper two treatment arms, and that 50% of conversions would be detected by MRI. They indicated that a sample size of 240 (60 per arm) would provide 80% power at two-sided α = 0.05.

#### Primary intention to treat analyses

**Figure 1:**
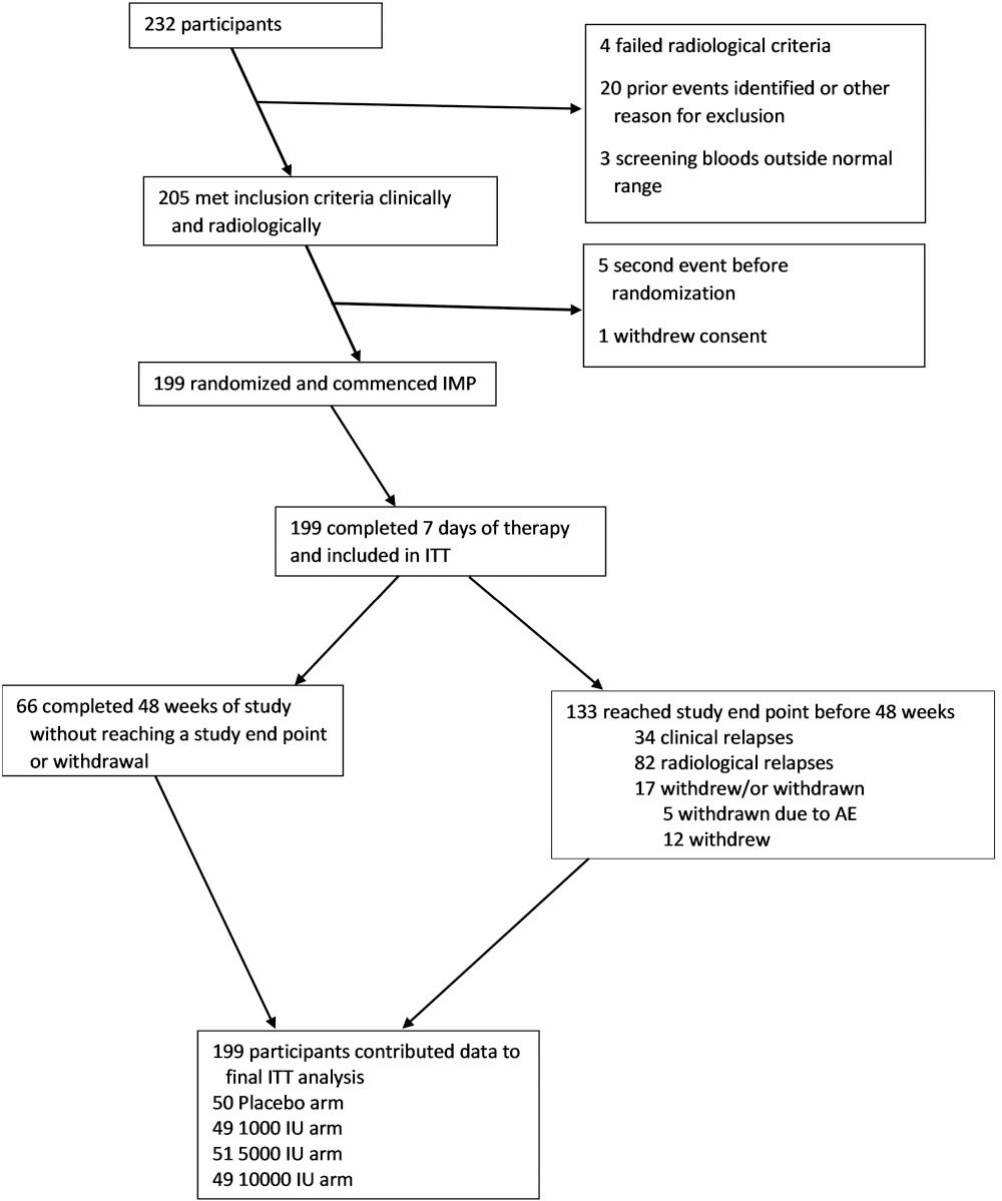
**PREVANZ study, flow chart of participants.** AE = adverse event; IMP = Investigational Medicinal Product; ITT = intention to treat; IU = international units.

Participants who took at least seven doses of study medication qualified for the intention to treat (ITT) analysis; other participants were omitted (Fig. 1). A first model compared the primary outcome (either clinical or radiological relapse) between the four randomized treatment arms without adjustment for other factors. In a second model, we included adjustment for age, sex and study centre (either as a categorical variable or according to numerical latitude of the site).

#### Additional analyses

We tested baseline values of known risk factors against conversion to MS after a CIS, including number of symptoms at CIS (≤1 versus ≥2), CIS onset site (optic nerve, brainstem/cerebellum, spinal cord or cerebrum); MRI lesion load (≥9 versus < 9 T_2_ lesions); presence/absence of infratentorial lesions; baseline EDSS; and smoking status. We included relevant variables in a further adjusted model.

We explored further adjustment for skin colour, season of enrolment in the study, BMI and baseline serum 25(OH)D_3_ level, on the outcome. A final fully adjusted model included any identified significant predictors.

In sensitivity analyses, we assessed the effects of (i) restricting the cohort to those who had a baseline serum 25(OH)D < 50 nmol/l (vitamin D deficient) and to <30 nmol/l (severely vitamin D deficient); and (ii) restricting the cohort to those who had completed at least 30 and 60 days on the trial to assess the effects of early conversion (biologically unlikely to be influenced by commencing supplementation) and early withdrawal on study outcomes.

Finally, we further explored the role of latitude and/or study centre in the model, i.e. whether the effects of vitamin D supplementation on the outcome varied according to latitude. A major source of vitamin D_3_ additional to supplementation is sunlight exposure, with levels of UV radiation varying inversely according to latitude. Our study centres in Australia and New Zealand extended across a wide latitudinal span from 27.5° (Brisbane, Australia) to 46.5° (Dunedin, New Zealand) South, and there is a well described latitudinal gradient of MS incidence and prevalence across this range in both countries.^10^ As we specifically randomized doses in blocks of four within centre, participants were (as nearly as possible) balanced across the four doses. Thus, our model estimates are, by design, within centre (and within region) estimates. Additional models fitted latitude as a continuous variable to test the effect of latitude on conversion rate and whether there was any dose-effect modification by latitude.

### Statistical analysis

The main objective of the study was assessed using proportional hazards interval-censored survival analysis (using the ‘stintreg’ command in Stata). The event of interest was either a confirmed clinical relapse and/or a new brain MRI T_2_ lesion (radiologically detected relapse). The dependent variable was the time in days from enrolment, either to first relapse of either type, or to the censoring date. For participants in whom relapse was detected radiologically, the censoring intervals were approximately either (0, 24) or (24, 48) weeks, calculated in days from enrolment. For participants in whom no relapse was recorded, the censoring date was the date of the last MRI.

## Results

Between 2013 and 2019 we enrolled 204 participants into the trial from 23 centres in Australia and New Zealand (Fig. 1); of these 199 were eligible for the ITT analysis. Table 1 shows the demographic and baseline characteristics of the four intervention groups and overall.

**Table 1:** Characteristics of participants in the PREVANZ clinical trial

	Placebo	1000 IU/day	5000 IU/day	10 000 IU/day	Overall
*n*	50	49	51	49	199
M:F	12 : 38	16 : 33	14 : 37	15 : 34	57 : 142
Age, mean (SD)	35.9 (9.8)	38.3 (10.3)	36.5 (10.6)	37.3 (10.6)	37.0 (10.3)
Baseline 25(OH)D_3_ level, nmoles/l, mean (SD)	71.2 (39.8)	65.8 (23.3)	69.9 (28.0)	69.9 (21.0)	69.0 (29.0)
Baseline 25(OH)D_3_ < 50 nmol/l, *n* (%)	15 (30)	14 (29)	12 (24)	7 (14)	48 (24)
Baseline 25(OH)D_3_ < 30 nmol/l, *n* (%)	4	1	4	1	10 (5)
Current smoker, *n* (%)	8 (16)	9 (18)	4 (8)	8 (17)	29 (14.6)
BMI kg/m^2,^ mean (SD)	29.2 (6.9)	29.3 (6.3)	29.5 (8.8)	27.3 (6.2)	28.8 (7.1)
Number of MRI T_2_ lesions, < 9 : ≥ 9	37: 13	33: 16	29 : 22	36: 13	135 : 64
Infratentorial lesions, 0: ≥ 1	30 : 20	25 : 24	33 : 18	31 : 18	119 : 80
Given steroids at onset, *n* (%)	27 (54)	27 (55)	31 (61)	26 (53)	111 (56)
Number of baseline symptoms, mean (SD)	1.88 (1.17)	1.63 (1.13)	1.71 (1.24)	1.90 (0.98)	1.78 (1.13)
Converted to MS clinically, *n* (%)	10 (20)	5 (10)	9 (18)	10 (20)	34 (68)
Converted to MS radiologically, *n* (%)	17 (34)	20 (40)	24 (47)	21 (42)	82 (42)
Withdrew without conversion: *n* (mean days on study)	5 (167)	4 (69)	2 (81)	1 (20)	12 (108)
Withdrawn due to AE/pregnancy, *n*	3	0	2	0	5
Did not convert, *n* (%)	15 (30)	19 (39)	15 (29)	17 (35)	66 (33)
Converted to MS 24 weeks, *n* (%)	22 (44)	21 (43)	26 (51)	23 (49)	92 (46)
Converted to MS 48 weeks, *n* (%)	27 (54)	25 (51)	33 (65)	31 (63)	116 (58)

AE = adverse event; BMI = body mass index; SD = standard deviation.

### Cohort retention and treatment compliance

Twelve people withdrew consent (6%), all before conversion, and five were withdrawn: four due to unplanned pregnancy and one to an AE (2%). One hundred and eighty-two people completed the study (91.5%) (Table 1 and Fig. 1).

**Figure 2:**
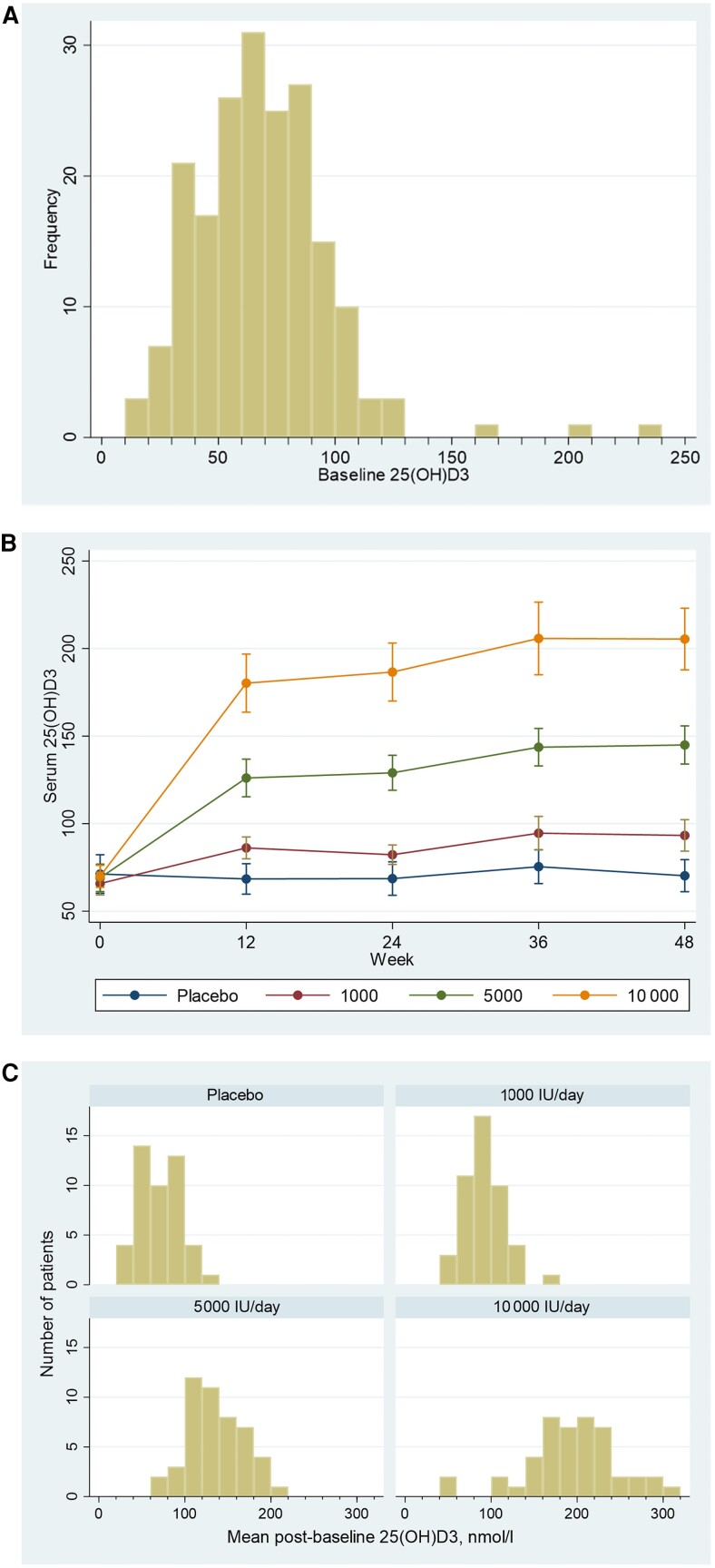
**Baseline and within study achieved serum 25(OH)D levels**. (**A**) Distribution of baseline serum 25(OH)D_3_ levels (nmol/l). (**B**) Mean serum 25(OH)D_3_ (nmol/l) levels by treatment group/dose and week (with 95% confidence intervals, CIs). (**C**) Mean serum 25(OH)D_3_ concentrations over the 48-week study period post baseline for all trial arms.

Figure 2 shows the baseline 25(OH)D_3_ levels for all participants (Fig. 2A), the change in mean 25(OH)D_3_ levels across treatment groups over the course of the trial (Fig. 2B), and the mean post-baseline (up to four time points) serum 25(OH)D_3_ concentrations for each trial arm (Fig. 2C). As expected, there was a dose-response in the 25(OH)D_3_ level according to dose of vitamin D_3_ supplementation; mean 25(OH)D_3_ levels remained very similar within each group from 12–48 weeks (Fig. 2C) and are consistent with a relatively uniform uptake of vitamin D supplementation and very rare instances of potential non-compliance or unreported supplementation.

### Intention to treat analysis

**Figure 3:**
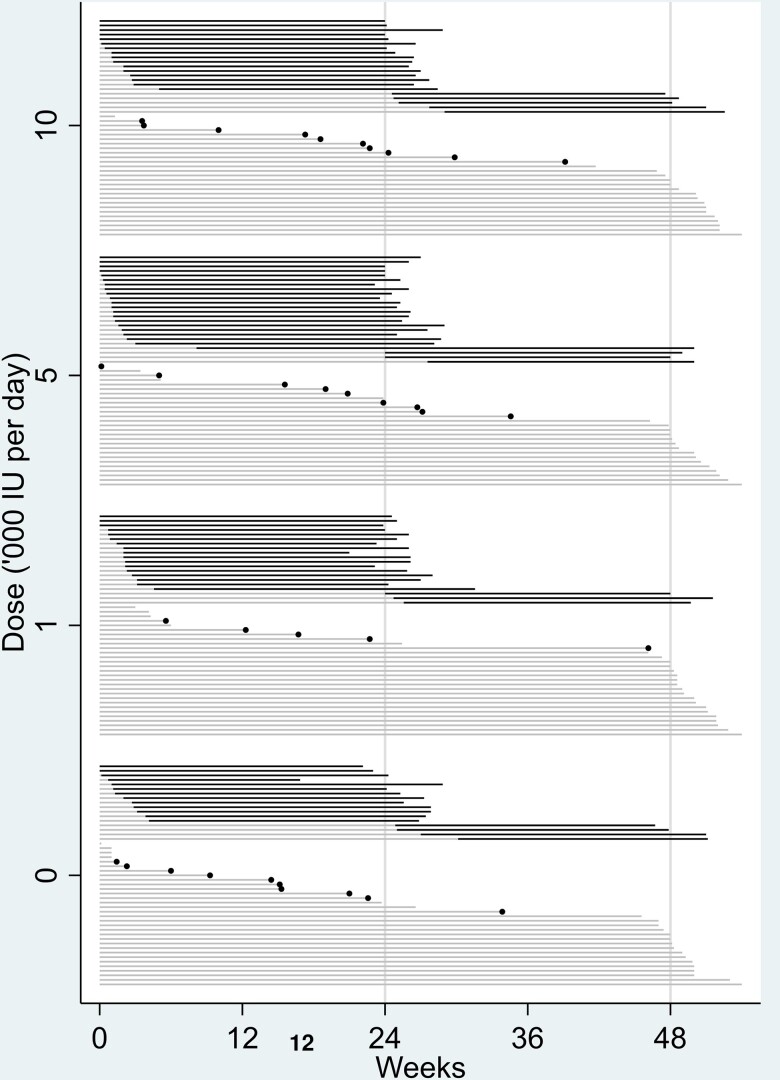
**Outcomes for all participants by assigned treatment**. MRI conversions plotted above clinical conversions and right-censored (non-converted) participants, and then plotted in order of failure time. Grey lines show time on study without conversion. Short light grey lines ending in a black dot show clinical conversions. Dark grey lines show intervals between MRI scans during which radiological conversion took place. IU = international units.

Figure 3 shows the outcomes for all participants by assigned treatment arm. In the unadjusted model, we found no evidence of an effect of vitamin D_3_ supplementation relative to placebo [hazard ratios (HRs) (95% confidence intervals, CIs): 1000 IU/day 0.87 (0.50, 1.50); 5000 IU/day 1.37 (0.82, 2.29); 10 000 IU/day 1.28 (0.76, 2.14)] on risk of conversion to MS. There was no overall effect of the four treatments, *P* = 0.28.

We tested the effect of other factors on the outcome. There was no effect of sex (*P* = 0.42), but a linear dependence on age (*P* = 0.002), with HR = 0.97, 95% CI 0.95, 0.99, per 1 year increase in age. There was no curvature in this relationship (*P* = 0.96). The variation in HRs between centres was significant (*P* = 0.008), with HRs relative to AU-01 (Royal Melbourne Hospital) ranging from 0.45 to 7.33. After adjusting for these variables, we did not find evidence of superiority of vitamin D_3_ supplementation versus placebo in reducing the risk of conversion to MS [1000 IU/day 0.79 (0.44, 1.41), 5000 IU/day 1.15 (0.67, 1.99) 10 000 IU/day 1.06 (0.61, 1.83)]. There was no overall effect of the four treatments, *P* = 0.60.

### Effect of other baseline factors on risk of conversion

There was no overall association between smoking and the risk of conversion. Other factors measured at baseline, including skin colour, BMI, season of enrolment in the study, disability level (EDSS score), use of glucocorticoids at CIS and the site of CIS did not influence the risk of conversion to MS during the study period (all *P* > 0.10).

Polysymptomatic versus monosymptomatic CIS was associated with a higher risk of conversion to MS [HR = 1.22 (CI 1.02, 1.45) for each additional symptom, *P* = 0.027] but there was no interaction with vitamin D_3_ dose (*P* = 0.75). Having one or more infratentorial lesions on the baseline MRI increased the HR for conversion to 1.82 (CI 1.16–2.86), but there was no interaction with dose (*P* = 0.86).

The number of baseline cerebral MRI T_2_ lesions was not associated with conversion to clinically definite or radiological MS (*P* = 0.08).

In a fully adjusted model including age, sex, study centre, symptom number and number of infratentorial lesions, the adjusted HRs (compared to placebo) were: 1000 IU/day 0.80 (0.45, 1.44); 5000 IU/day 1.36 (0.78, 2.38); 10 000 IU/day 1.07 (0.62, 1.85). There was no overall effect of the four treatments (*P* = 0.34).

When restricting the analysis to those with 25(OH)D_3_ levels <50 nmol/l or <30 nmol/l, there remained no evidence for an association of vitamin D supplementation dose with conversion to MS, noting very low numbers in both analyses. Additionally, removing those who converted before 30 or 60 days did not alter the findings (data not shown).

### Analysis of the effect of study centre and latitude

We further explored the variation in HRs according to study centre noted above. We restricted the analysis to include only study centres that had recruited more than four participants (four centres in New Zealand and 10 in Australia), as randomization was within study centres and thus includes at least one participant in each study arm. In a model including latitude and recruitment size (both as 2df splines) and adjusted for age, sex and treatment allocation, the latitude effect remained statistically significant (*P* = 0.03) but with no evidence for an effect of study centre (*P* = 0.10) and no interaction between vitamin D_3_ dose and latitude (*P* = 0.91), or vitamin D_3_ dose and number of participants (*P* = 0.25).

**Figure 4:**
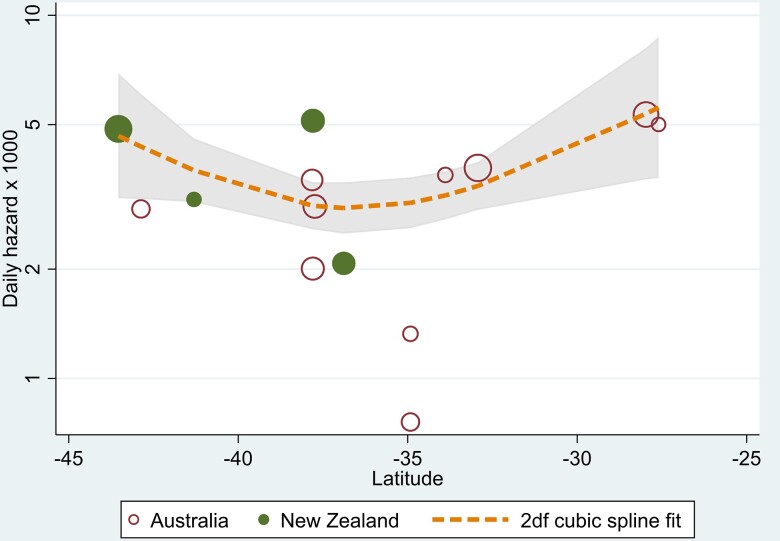
**Centre-specific risk of conversion per day on study**. Centre-specific risk of conversion per day on study, limited to those centres with more than four participants recruited. The symbol sizes (areas) are proportional to the number of participants accrued.

Figure 4 shows how risk of conversion (log scale) varies with latitude in degrees south. A model that fitted different curves in each country was not superior to the model including latitude and recruitment size as 2df splines.

Overall, these models did not show a meaningful effect of latitude or, by proxy, study centre on the risk of conversion, rather they showed that the differences were driven by centres at the mid-latitude points with lower conversion rates, i.e. there was no latitudinal gradient. Overall, latitude or study centre did not modify the association between vitamin D_3_ supplementation and conversion from CIS to MS.

### Safety outcomes

Table 2 displays the number of AEs and serious AEs (SAEs) seen in the trial by treatment assignment. AEs were graded by the principal investigators as mild, moderate or severe.

**Table 2:** The number of adverse events and serious adverse events seen in the trial by treatment assignment

	Placebo *n* = 50	1000 IU/day *n* = 49	5000 IU/day *n* = 51	10 000 IU/day *n* = 49	Total *n* = 199
AEs, *n* (%)	195 (28)	154 (22)	174 (25)	174 (25)	697
SAEs, *n* (%)	2 (15)	5 (38)	2 (15)	4 (30)	13 (2)
AEs, grading^a^, *n* (%)					
Mild	120(62)	76 (49)	100 (57)	105 (60)	401 (58)
Moderate	74 (38)	76 (49)	67 (39)	67 (39)	284 (41)
Severe	1 (1)	2 (1)	7 (4)	2 (1)	12 (2)
Association with therapy, *n* (%)					
Not/unlikely	183 (93)	150 (97)	166 (95)	171 (98)	670(96)
Possible	11 (6)	4 (3)	3 (2)	2 (1)	20 (3)
Probable	0	0	5 (3)	1 (1)	6 (1)
Definite	1 (1)	0	0	0	1 (0)
Study drug discontinued, *n*	3	0	2	0	5

AE = adverse event; SAE = serious adverse event.

^a^AEs graded by site primary investigator.

Overall vitamin D_3_ supplementation was well tolerated with 13 SAEs recorded, defined by hospitalizations. None of these were thought to be related to the study medication. There were two MS relapses requiring admission. There were no deaths recorded. Five participants had their study medication ceased: three in the placebo group (one due to raised calcium thought to be linked to the study medication, and two unplanned pregnancies) and two unplanned pregnancies in the 5000 IU group. We found no evidence of differences in the number and severity of AEs between the treatment groups.

The 13 SAEs recorded were: placebo: choking episode, major depression; 1000 IU/day: exacerbation of chronic abdominal pain, road traffic accident, asymptomatic embolic stroke (new imaging finding), major depression, kidney stone (normal Ca^2+^ and urate); 5000 IU/day: MS relapse, palpitations; 10 000 IU/day: MS relapse, perianal abscess, foot pain, caecal volvulus. Importantly there were no abnormalities detected on routine screening for hypercalcaemia, alteration in renal function or elevated urate levels in the active treatment arms.

## Discussion

We conducted a phase IIb double-blind, placebo-controlled, trial of three doses of oral vitamin D_3_ (cholecalciferol) compared to placebo. The end point was conversion to definite MS after a high risk CIS. In unadjusted and adjusted ITT analyses, doses of 1000 IU/day, 5000 IU/day and 10 000 IU/day of oral vitamin D_3_ were not associated with a reduced risk of conversion to definite MS either clinically (defined by validated relapse) or radiologically (defined by the appearance of a new MRI brain T_2_ lesion). Our study provides evidence that oral vitamin D_3_ supplementation does not have a beneficial effect on the risk of conversion to definite MS in a high-risk CIS group.

This trial was originally designed to assess whether vitamin D supplementation after a high-risk CIS (abnormal MRI) prevented conversion to definite MS based on the established diagnostic criteria at the time (McDonald, 2010)^28^. Subsequently with the evolution of diagnostic criteria (McDonald criteria, 2017)^29^ and a change in views regarding when MS starts in the biological context, it now seems appropriate to consider that the vast majority (but not all) of CIS cases (particularly those with abnormal MRIs as required to participate in this trial) already have MS and therefore our results can also be considered as assessing the effects of vitamin D supplementation on MRI and clinical disease activity in early MS.

These findings are consistent with thre recent meta-analyses of vitamin D supplementation trials in people with relapsing-remitting MS.^23-25^ However, they are discordant with Mendelian randomization studies that suggest that genetically determined higher 25(OH)D levels are causally related to lower MS risk,^17^ and CIS cohort studies such as the AusLong Study, undertaken over a similar latitudinal range, where lower past sun exposure was an independent risk factor for conversion from CIS to CDMS.^8,33^

Our study also had divergent findings from the only prior CIS monotherapy study, a small pilot study (*n* = 30) conducted in a single Iranian centre.^26^ This study randomized cases with a first episode of optic neuritis (CIS) who were vitamin D deficient (<30 nmol/l) to placebo or high-dose vitamin D supplementation (50 000 IU/week) in a 1:1 ratio. Thirty participants were enrolled and 24 completed the 48-week study, which also included follow-up MRI at 24 and 48 weeks. The study found a significant benefit of vitamin D supplementation on relapse and MRI disease activity. As our study population was not vitamin D deficient at baseline, it is possible that vitamin D supplementation in vitamin D deficient people with CIS could still have a therapeutic effect. However, when we assessed separately those with a lower baseline vitamin D level (<50 nmol/l or <30 nmol/l) there remained no association between vitamin D_3_ supplementation and the hazard of conversion to MS, although numbers were low [*n* = 10 with 25(OH)D_3_ levels < 30 nmol/l]. We set out to determine whether vitamin D monotherapy commenced after a high-risk CIS could modulate subsequent MS disease activity. Additionally, we elected not to limit eligibility to those with vitamin D deficiency, or indeed to measure 25(OH)D as part of assessment of eligibility, to maximize recruitment and to avoid having to exclude vitamin D sufficient participants. As can be seen from the results of this trial, if we had limited recruitment to those with vitamin D deficiency, we would profoundly limit recruitment, which would have made the trial infeasible. Similarly, when this trial was designed there was no clear evidence that vitamin D was a DMT in MS, despite it being used in this way by many people. We therefore sought to determine in our population whether vitamin D was useful as a DMT across the whole CIS population.

We considered multiple potential confounders in our analyses based on variables that have been associated with worse outcomes or greater risk of relapse in MS and CIS trials. These included age and sex, BMI, skin colour, treatment of the index attack with glucocorticoids, baseline MRI characteristics, particularly number of T_2_ lesions and presence of infratentorial lesions, number of MS symptoms, onset site of CIS, and disability level at baseline. We also considered study centre effects and the latitude of the study centres, as latitude is a proxy for ambient UV radiation, which could in turn relate to vitamin D status. We found that younger age, polysymptomatic onset and presence of infratentorial MRI lesions increased the risk of relapse, concordant with previous literature.^31,33^ We adjusted our ITT analysis for all these effects, but this did not alter the findings.

Study centre (and the latitude of the study centre) were associated with the probability of conversion to MS. However, this was not a north to south latitudinal gradient in risk of conversion. Explicitly modelling latitude as a continuous curve removed all latitude effects, and there was then no remaining residual effect of centre on the outcome, and no significant variation between centres in dose effects. Thus, we found that there was a main effect of latitude on conversion rate, but there was no modification of the dose-effect by latitude.

The study had some limitations. First, we were underpowered for our primary outcome as we were unable to recruit the 240 participants estimated by our power calculations to be required to show a 30% reduction in risk of conversion. We recruited 204 participants over a nearly 7-year period and elected to end the study due to very low ongoing recruitment levels. The principal barrier to recruitment came with the publication of the McDonald Criteria 2017,^29^ which changed the definition of MS to allow diagnosis at CIS either by radiological criteria or by detection of CSF-restricted oligoclonal bands, thus allowing earlier prescription of DMTs. However, the consistency of our findings across all models without any suggestion of a trend towards efficacy would suggest that the negative outcome is sufficiently supported by the data. Given the results, it is unlikely that a difference between arms would have been detected even if the recruitment target had been achieved. Second, we are unable to provide any information on the interactions of vitamin D_3_ and DMTs as we required all participants to delay treatment with DMTs while in the trial until a second event occurred. Third, these results may not be generalizable to CIS with vitamin D deficiency at onset. Our study population was a replete population with the mean serum 25(OH)D_3_ level of 69 nmol/l and very few participants with levels below 30 nmol/l. Fourth, we employed a very strict definition of CIS to recruit a study population at high risk of conversion to definite MS within the study time period. Therefore, our results may not be generalizable to the whole CIS group with fewer or no MRI lesions. Undertaking MS prevention trials after CIS is no longer a feasible option due to the changes in diagnostic criteria. True prevention trials at the population level in low ambient UV locations in at-risk populations could be considered but there are multiple logistical concerns that would make this type of trial difficult to undertake. Critically, when such an intervention should be targeted is problematic as an association between increased MS risk and low vitamin D/low sunlight exposure has been described from gestation,^15^ to prior to CIS onset^7^; often more than 30 years later. Similarly, trials at putative earlier stages of MS, radiologically isolated syndrome or MS prodrome would also be technically difficult, particularly regarding recruitment and the length of time required to define a robust outcome. Finally, it may be that serum 25(OH)D levels in previous observational studies may simply be a proxy for sunlight exposure, as it has been shown that sun exposure can act independently of vitamin D to reduce the risk of MS.^13^ In addition, the adverse effect of vitamin D deficiency on MS risk has been shown to be weaker in higher sun exposure conditions, with evidence of an additive interaction.^13^ Thus, findings from this Australian and New Zealand trial may not be generalizable to lower ambient UVR locations. Further work is required to test the possible effects of individual level sun exposure, other vitamin D metabolites, diet, individual level genetic factors, and other environmental exposures on outcomes in the PrevANZ study.

The strengths of this study include the strict application of only vitamin D_3_ or placebo oral therapy in a double-blind four-arm study that allowed us to study the effects of a range of vitamin D_3_ doses without confounding by DMT use. We recruited participants across a wide range of latitudes, thus allowing us to address the confounding effects of environment to some extent. We showed that the uptake of vitamin D_3_ supplementation was consistent across the arms and efficacious in elevating serum 25(OH)D_3_. Finally, recruitment was not influenced by baseline vitamin D status.

## Conclusion

This study does not support the use of oral vitamin D supplementation (across the dose range 1000 to 10 000 IU per day) in vitamin D replete adults following a CIS with three or more MRI CNS T_2_ lesions, to prevent conversion to clinically definite or radiological MS. Nor does it support the use of oral vitamin D at this dosage range as a DMT in early MS.

## Supplementary Material

awad409_Supplementary_Data

## Data Availability

Data from this study are available from the data custodians MS Australia to *bona fide* researchers on completion of a research collaboration agreement available from MSA.org.au and on approval of the MSA constituted PREVANZ Data access committee.

## References

[1] McGinley MP , Goldschmidt CH, Rae-Grant AD. Diagnosis and treatment of multiple sclerosis: A review. JAMA. 2021;325:765–779. 33620411 10.1001/jama.2020.26858

[2] Thompson AJ , Baranzini SE, Geurts J, Hemmer B, Ciccarelli O. Multiple sclerosis. Lancet. 2018;391:1622–1636. 29576504 10.1016/S0140-6736(18)30481-1

[3] Zarghami A , Li Y, Claflin SB, van der Mei I, Taylor BV. Role of environmental factors in multiple sclerosis. Expert Rev Neurother. 2021;21:1389–1408. 34494502 10.1080/14737175.2021.1978843

[4] International Multiple Sclerosis Genetics Consortium . Multiple sclerosis genomic map implicates peripheral immune cells and microglia in susceptibility. Science. 2019;365:eaav7188. 31604244 10.1126/science.aav7188 PMC7241648

[5] Olsson T , Barcellos LF, Alfredsson L. Interactions between genetic, lifestyle and environmental risk factors for multiple sclerosis. Nat Rev Neurol. 2017;13:25–36. 27934854 10.1038/nrneurol.2016.187

[6] Waubant E , Lucas R, Mowry E, et al Environmental and genetic risk factors for MS: An integrated review. Ann Clin Transl Neurol. 2019;6:1905–1922. 31392849 10.1002/acn3.50862 PMC6764632

[7] Lucas RM , Lucas RM, Ponsonby A-L, et al Sun exposure and vitamin D are independent risk factors for CNS demyelination. Neurology. 2011;76:540–548. 21300969 10.1212/WNL.0b013e31820af93d

[8] Simpson S Jr , van der Mei I, Lucas RM, et al Sun exposure across the life course significantly modulates early multiple sclerosis clinical course. Front Neurol. 2018;9:16. 29449827 10.3389/fneur.2018.00016 PMC5799286

[9] Simpson S Jr , Blizzard L, Otahal P, Van der Mei I, Taylor B. Latitude is significantly associated with the prevalence of multiple sclerosis: A meta-analysis. J Neurol Neurosurg Psychiatry. 2011;82:1132–1141. 21478203 10.1136/jnnp.2011.240432

[10] Simpson S Jr , Wang W, Otahal P, Blizzard L, van der Mei IAF, Taylor BV. Latitude continues to be significantly associated with the prevalence of multiple sclerosis: An updated meta-analysis. J Neurol Neurosurg Psychiatry. 2019;90:1193–1200. 31217172 10.1136/jnnp-2018-320189

[11] Sebastian P , Cherbuin N, Barcellos LF, et al Association between time spent outdoors and risk of multiple sclerosis. Neurology. 2022;98:e267–e278. 34880094 10.1212/WNL.0000000000013045 PMC8792813

[12] Hedström AK , Hillert J, Olsson T, Alfredsson L. Factors affecting the risk of relapsing-onset and progressive-onset multiple sclerosis. J Neurol Neurosurg Psychiatry. 2021;92:1096–1102. 33986119 10.1136/jnnp-2020-325688 PMC8458089

[13] Hedström AK , Olsson T, Kockum I, Hillert J, Alfredsson L. Low sun exposure increases multiple sclerosis risk both directly and indirectly. J Neurol. 2020;267:1045–1052. 31844981 10.1007/s00415-019-09677-3 PMC7109160

[14] Munger KL , Levin LI, Hollis BW, Howard NS, Ascherio A. Serum 25-hydroxyvitamin D levels and risk of multiple sclerosis. JAMA. 2006;296:2832–2838. 17179460 10.1001/jama.296.23.2832

[15] Jasper EA , Nidey NL, Schweizer ML, Ryckman KK. Gestational vitamin D and offspring risk of multiple sclerosis: a systematic review and meta-analysis. Ann Epidemiol. 2020;43:11–17. 32014337 10.1016/j.annepidem.2019.12.010

[16] Nielsen NM , Munger KL, Koch-Henriksen N, et al Neonatal vitamin D status and risk of multiple sclerosis: A population-based case-control study. Neurology. 2017;88:44–51. 27903815 10.1212/WNL.0000000000003454 PMC5200855

[17] Wang R . Mendelian randomization study updates the effect of 25-hydroxyvitamin D levels on the risk of multiple sclerosis. J Transl Med. 2022;20:3. 34980161 10.1186/s12967-021-03205-6 PMC8722044

[18] Sabel CE , Pearson JF, Mason DF, Willoughby E, Abernethy DA, Taylor BV. The latitude gradient for multiple sclerosis prevalence is established in the early life course. Brain. 2021;144:2038–2046. 33704407 10.1093/brain/awab104

[19] Simpson S Jr , der Mei IV, Taylor B. The role of vitamin d in multiple sclerosis: Biology and biochemistry, epidemiology and potential roles in treatment. Med Chem. 2018;14:129–143. 28933265 10.2174/1573406413666170921143600

[20] Soilu-Hänninen M , Åivo J, Lindström B-M, et al A randomised, double blind, placebo controlled trial with vitamin D3 as an add on treatment to interferon beta-1b in patients with multiple sclerosis. J Neurol Neurosurg Psychiatry. 2012;83:565–571. 22362918 10.1136/jnnp-2011-301876

[21] Stein MS , Liu Y, Gray OM, et al A randomized trial of high-dose vitamin D2 in relapsing-remitting multiple sclerosis. Neurology. 2011;77:1611–1618. 22025459 10.1212/WNL.0b013e3182343274

[22] Hupperts R , Smolders J, Vieth R, et al Randomized trial of daily high-dose vitamin D(3) in patients with RRMS receiving subcutaneous interferon beta-1a. Neurology. 2019;93:e1906–e1916. 31594857 10.1212/WNL.0000000000008445 PMC6946471

[23] Jagannath VA , Filippini G, Borges do Nascimento IJ, Di Pietrantonj C, Robak EW, Whamond L. Vitamin D for the treatment of multiple sclerosis: A meta-analysis. J Neurol. 2018;265:2893–2905. 30284038 10.1007/s00415-018-9074-6

[24] Jagannath VA , Filippini G, Borges do Nascimento IJ, Di Pietrantonj C, Robak EW, Whamond L. Vitamin D for the management of multiple sclerosis. Cochrane Database Syst Rev. 2018;9:CD008422. 30246874 10.1002/14651858.CD008422.pub3 PMC6513642

[25] Zheng C , He L, Liu L, Zhu J, Jin T. The efficacy of vitamin D in multiple sclerosis: A meta-analysis. Mult Scler Relat Disord. 2018;23:56–61. 29778041 10.1016/j.msard.2018.05.008

[26] Derakhshandi H , Etemadifar M, Feizi A, et al Preventive effect of vitamin D3 supplementation on conversion of optic neuritis to clinically definite multiple sclerosis: A double blind, randomized, placebo-controlled pilot clinical trial. Acta Neurol Belg. 2013;113:257–263. 23250818 10.1007/s13760-012-0166-2

[27] Kappos L , Freedman MS, Polman CH, et al Effect of early versus delayed interferon beta-1b treatment on disability after a first clinical event suggestive of multiple sclerosis: A 3-year follow-up analysis of the BENEFIT study. Lancet. 2007;370:389–397. 17679016 10.1016/S0140-6736(07)61194-5

[28] Polman CH , Reingold SC, Banwell B, et al Diagnostic criteria for multiple sclerosis: 2010 revisions to the McDonald criteria. Ann Neurol. 2011;69:292–302. 21387374 10.1002/ana.22366 PMC3084507

[29] Thompson AJ , Banwell BL, Barkhof F, et al Diagnosis of multiple sclerosis: 2017 revisions of the McDonald criteria. Lancet Neurol. 2018;17:162–173. 29275977 10.1016/S1474-4422(17)30470-2

[30] Miller D , Barkhof F, Montalban X, Thompson A, Filippi M. Clinically isolated syndromes suggestive of multiple sclerosis, part 2: non-conventional MRI, recovery processes, and management. Lancet Neurol. 2005;4:341–348. 15907738 10.1016/S1474-4422(05)70095-8

[31] Tintoré M , Rovira A, Río J, et al Baseline MRI predicts future attacks and disability in clinically isolated syndromes. Neurology. 2006;67:968–972. 17000962 10.1212/01.wnl.0000237354.10144.ec

[32] Paty DW , Oger JJF, Kastrukoff LF, et al MRI in the diagnosis of MS: A prospective study with comparison of clinical evaluation, evoked potentials, oligoclonal banding, and CT. Neurology. 1988;38:180–185. 3340277 10.1212/wnl.38.2.180

[33] Chapman C , Lucas RM, Ponsonby A-L, et al Predictors of progression from a first demyelinating event to clinically definite multiple sclerosis. Brain Commun. 2022;4:fcac181. 35891671 10.1093/braincomms/fcac181 PMC9308470

