# Supplementary material for: Vitamin D did not reduce multiple sclerosis disease activity after a clinically isolated syndrome
Source: Brain. 2023 Dec 12;147(4):1206–15. doi: 10.1093/brain/awad409 (PMC10994527; doi:10.1093/brain/awad409)
Supplement: awad409_Supplementary_Data [file awad409_supplementary_data.zip › brain-2023-01579-File008.pdf]

## Supplementary Materials

### Section 1 Randomisation schedule

Participants were randomized to individual study arms using the eCRF REDCap system, stratified by study sites, and using randomly permuted blocks of size 4 to maintain sequential balance. Block size was not disclosed to the investigators until the study data lock.

| | |
|---|---|
| Randomization schedule for Vitamine D study | |
| Created on: | 23-Jan-13 |
| Created by: | Prof L.Churilov, Florey Institute of Neuroscience and Mental Health, e-mail: leonid.churilov@florey.edu.au |
| Notes: | a) There are four arms of the study: 0, 1, 5, and 10 K units - in the schedule denoted respectively as 0,1,5, and 10 |
| | b) There are 240 patients |
| | c) There are 21 sites participating in the study - denoted as Site_X in the workbook |
| | d) Permuted blocks of four are used to ensure tight balance as per the request of the NTA |
| | e) As there is no certainty about the anticipated recruitment levels for each site, the schedule is created so that each site can potentially recruit up to the full sample size of 240 patients. |
| | f) "Formulas" worksheet provides the details for populating the columns other than the random number column. The contents are then copied and pasted across individual worksheets using "values only" option. |
| | g) Uniformly distributed random numbers are generated using a site-specific seed and then the columns B,C, and D are sorted first by block number, and then by the random value in increasing order. |
| | h) Columns B and D are subsequently hidden to improve clarity (ie Block number and random number) |
| | |

**Section 2 inclusion and Exclusion Criteria**

***Inclusion criteria***

- aged between 18 and 65 years old inclusive.
- have a first isolated, well-defined, uni- or multi-focal first demyelinating event (FDE)
- be able to receive first dose of study drug within 120 days of FDE symptom onset.
- have an MRI brain scan that is supportive of demyelinating disease (Paty A or Paty B criteria).
- an EDSS between 0 - 6.5 (inclusive)
- be able to give informed consent and sign the informed consent form.
- willing to avoid open-label vitamin D supplementation and external serum vitamin D testing for the duration of the study.
- not have received any prior disease modifying treatment for MS other than corticosteroids.
- be able and willing to comply with all study procedures including MRI scanning as per protocol.
- if female of child-bearing age, must be willing to use effective contraceptive methods for the duration of the study.

***Exclusion Criteria***

Any of the following conditions will exclude a participant from the study:

- Age less than 18 years or greater than 65 years.
- Pregnant or nursing females.
- EDSS greater than 6.5.
- A documented or likely prior neurological event consistent with demyelination.
- Treatment with beta-interferon, glatiramer acetate, natalizumab, mitoxantrone, or other chemotherapeutic agent specifically for demyelinating disease.
- A second clinical demyelinating event prior to randomisation.
- A history of hypercalcaemia or primary hyperparathyroidism.
- A history of sarcoidosis.
- A history of renal calculi.
- A history of any condition requiring treatment with calcium, vitamin D or bisphosphonates.
- Hypercalcaemia on screening blood tests.

- An abnormal eGFR (<60 ml/min/1.73m2), or an elevated uric acid laboratory value (above normal range for the local laboratory used).
- Concurrent diagnosis of other neurological, psychiatric or other disease which, in the opinion of the investigator, could impair capacity to provide informed consent, interfere with study compliance, or impair the participant's ability to comply with the study protocol.
- Any contraindication to MRI scanning or intravenous Gadolinium including:
    - Cardiac Pacemaker
    - Cardiac Defibrillator
    - Any other non-MRI compatible medical device/implant
    - Previous reaction to Gadolinium
    - Severe claustrophobia

**Section 3 Study protocol**

| Item | Screen | Baseline | Wk 4 | Wk 12 | Wk 24 | Wk 36 | Wk 48 |
|---|---|---|---|---|---|---|---|
| Consent | ● | | | | | | |
| Eligibility Check List | ● | ● | | | | | |
| **Clinical** | | | | | | | |
| Relapse History | ● | ● | ● | ● | ● | ● | ● |
| Past History | ● | | | | | | |
| KFS, EDSS | ● | ● | | ● | ● | ● | ● |
| MSFC | ● | ● | | ● | ● | ● | ● |
| Review Clinical MRI | ● | | | | | | |
| Adverse Event History | | | ● | ● | ● | ● | ● |
| Concomitant Medications | ● | ● | | ● | ● | ● | ● |
| Medication Pill Count | | | | ● | ● | ● | ● |
| Quality of life survey | | ● | | ● | ● | | ● |
| Fatigue Score | | ● | | ● | ● | | ● |
| Depression Score | | ● | | ● | ● | | ● |
| **Laboratory** | | | | | | | |
| Serum pregnancy Test | ● | | | | | | |
| Urine Pregnancy Test | | | ● | ● | ● | ● | ● |
| Creatinine, Uric Acid, Ca | ● | | ● | ● | ● | ● | ● |
| Serum for storage (Vitamin D) | ● | | | ● | ● | ● | ● |
| **Imaging** | | | | | | | |
| Study MRI | ● | | | | ● | | ● |

**Section 4 MRI methods**

MRI scanning was performed at 20 sites using either 3T or 1.5T systems. Scans were obtained at baseline, week 24 and week 48. For each visit, the scanning protocol consisted of 2D dual-echo T2, FLAIR and pre- and post-gadolinium enhanced T1 weighted sequences in the transverse plane, using contiguous 3mm slices. Images were reconstructed to provide in-plane resolution of 1x1mm. A pre-contrast 3D T1 weighted sequence was acquired with a reconstructed voxel size of 1x1x1 mm.

All scans were reviewed on arrival to confirm compliance with the trial MRI acquisition protocol and for image quality. Deviation from the protocol or image quality issues (for instance presence of significant artifacts) resulted in the examination or part of the examination being rejected and not included in the analysis or a particular element of the analysis.

In addition, with respect to the whole brain volume measurement, subjects were excluded from this analysis if there had been a change of MRI scanner at the imaging site during the trial. A small number of subjects were also not included if final quality checks identified processing failures with respect to the brain atrophy analysis pipeline."

All gadolinium enhancing lesions present at the baseline visit were marked and counted. Numbers of new gadolinium enhancing and new T2 lesions were identified by visual inspection at weeks 24 and 48. Volumetric lesion analysis was performed by experienced operators using JIM v7 software (Xinapse systems, Aldwincle, UK). T2 lesions were segmented at baseline to provide a measure of T2 lesion volume. For each subject the baseline T2 lesion masks were transposed to the week 48 scans which were registered to the corresponding baseline scan. T2 lesion masks were edited to reflect any changes observed on the week 48 visit.

The processing pipeline used for volume and atrophy measurements started with N4 bias field correction [1] of the 3DT1 image using ANTs. The T2 image was then registered to the T1 and this transform was applied to the associated lesion mask using niftyreg ([2}.The registered lesion mask was then used to fill lesions on the 3DT1 using an in-painting method [3}. The 3DT1 image was then segmented using the Geodesic Information Flows algorithm [4]. At baseline, brain volume was measured using SIENAX[5]) from FSL [6]. Atrophy was measured from baseline to week 48 using SIENA (Smith et al., 2002) also from FSL.

1 Tustison, N.J., Avants, B.B., Cook, P.A., Zheng, Y., Egan, A., Yushkevich, P.A., Gee, J.C., 2010. N4ITK: improved N3 bias correction. IEEE Trans. Med. Imaging 29, 1310–20. https://doi.org/10.1109/TMI.2010.2046908

2 Modat, M., Cash, D.M., Daga, P., Winston, G.P., Duncan, J.S., Ourselin, S., 2014. Global image registration using a symmetric block-matching approach. J. Med. Imaging 1, 024003. https://doi.org/10.1117/1.JMI.1.2.024003

3 Prados, F., Cardoso, M.J., Kanber, B., Ciccarelli, O., Kapoor, R., Gandini Wheeler-Kingshott, C.A.M., Ourselin, S., 2016. A multi-time-point modality-agnostic patch-based method for lesion filling in multiple sclerosis. NeuroImage 139, 376–384. https://doi.org/10.1016/J.NEUROIMAGE.2016.06.053

4 Cardoso, M.J., Modat, M., Wolz, R., Melbourne, A., Cash, D., Rueckert, D., Ourselin, S., 2015. Geodesic Information Flows: Spatially-Variant Graphs and Their Application to Segmentation and Fusion. IEEE Trans. Med. Imaging 34, 1976–1988. https://doi.org/10.1109/TMI.2015.2418298

5 Smith, S.M., Jenkinson, M., Woolrich, M.W., Beckmann, C.F., Behrens, T.E.J., Johansen-Berg, H., Bannister, P.R., De Luca, M., Drobnjak, I., Flitney, D.E., Niazy, R.K., Saunders, J., Vickers, J., Zhang, Y.,

De Stefano, N., Brady, J.M., Matthews, P.M., 2004. Advances in functional and structural MR image analysis and implementation as FSL, in: NeuroImage. Academic Press, pp. S208–S219. https://doi.org/10.1016/j.neuroimage.2004.07.051

6 Smith, S.M., Zhang, Y., Jenkinson, M., Chen, J., Matthews, P.M., Federico, A., De Stefano, N., 2002. Accurate, robust, and automated longitudinal and cross-sectional brain change analysis. NeuroImage 17, 479–489. https://doi.org/10.1006/nimg.2002.1040

**Section 5 Vitamin D analysis methods**

**Method summaries for PREVANZ Trial 2021/22**

**Dr Michael Clarke (April 2022)**

**Method 1: 25(OH)D3, Epi-25(OH)D3 and 25(OH)D2.**

This method has been published and has been previously used for samples that have been assessed using the Vitamin D Standardisation Program (VDSP) managed by the Centre for Disease Control, USA. https://www.cdc.gov/labstandards/pdf/hs/CDC_Certified_Vitamin_D_Procedures-508.pdf

In brief, 50µL of serum was extracted into Hexane: Ethyl Acetate, reconstituted in 70% methanol and assayed using a 2D LC-MS/MS platform, described in detail elsewhere (1). The limit of quantitation (LOQ) for 25(OH)D3 and Epi-25(OH)D3 is 2.0 nM, for 25(OH)D2 the LOQ is 3 nM. Assay precision was assessed using quality controls (QCs) supplied UTAK™ with low, medium and high QC's assayed at the beginning and end of each batch. Precision for the cohort, assessed by coefficient of variation (CV%), was 1.9% (30.4 nM), 2.4% (79.1 nM) and 2.6% (179.7 nM). A separate QC material used was a pooled human serum sample containing endogenous 25(OH)D3 and this was spiked with Epi-25(OH)D3 and 25(OH)D2. Precision for 25(OH)D3 was 7.0% (45.3 nM), Epi-25(OH)D3 was 13.7% (24.4 nM) and for 25(OH)D2 was 12.6% (17.0 nM).


1. Clarke, M. W., et al. (2013). Optimized 25-hydroxyvitamin D analysis using liquid–liquid extraction with 2D separation with LC/MS/MS detection, provides superior precision compared to conventional assays. Metabolomics 9(5): 1031-1040. DOI: 10.1007/s11306-013-0518-9
2. Ding et al, (2010) Quantitative determination of Vitamin D metabolites in plasma using UHPLC-MS/MS. Anal Bioanal Chem 398:779–789. DOI 10.1007/s00216-010-3993-0
3. Tiller, C., et al. (2022). Vitamin D metabolites and risk of first clinical diagnosis of central nervous system demyelination. The Journal of Steroid Biochemistry and Molecular Biology 218: 106060. DOI: 10.1016/j.jsbmb.2022.106060

**Section 6 Consort Statement**

| Item Description | |
|---|---|
| **Title**<br>Identification of the study as randomised | Yes |
| **Trial design**<br>Description of the trial design | Yes |
| **Participants**<br>Eligibility criteria for participants and the settings | Yes |
| **Methods**<br>Where the data were collected | Yes |
| **Interventions**<br>Interventions intended for each group | Yes |
| **Objective**<br>Specific objective or hypothesis | Yes |
| **Outcome**<br>Clearly defined primary outcome | Yes |
| **Randomisation**<br>How participants were allocated to interventions | Yes |
| **Blinding**<br>Whether or not participants, care givers, and those assessing the outcomes were. blinded to group assignment | Yes |
| **Results** | |
| **Numbers** randomised. | Yes |
| **Number of participants** randomised to each. group. | Yes |
| **Recruitment** trial status | Yes |
| **Numbers** analysed | Yes |
| **Numbers** analysed in each group. | Yes |
| **Outcome** For the primary outcome, a r result for each group and the estimated effect size and its precision | Yes |
| **Harms** Important adverse events or side-effects | Yes |
| **Conclusions**<br>General interpretation of the result | |
| **Trial registration**<br>Registration number and name of trial register | Australian Clinical Trials Registry<br>ACTR12612001160820 |
| **Funding**<br>Source of funding | Fully funded by MS Australia |
