# Supplementary material for: Vitamin D did not reduce multiple sclerosis disease activity after a clinically isolated syndrome
Source: Brain. 2023 Dec 12;147(4):1206–15. doi: 10.1093/brain/awad409 (PMC10994527; doi:10.1093/brain/awad409)
Supplement: awad409_Supplementary_Data [file awad409_supplementary_data.zip › brain-2023-01579-File009.pdf]

# Vitamin D did not reduce multiple sclerosis disease activity after a clinically isolated syndrome

***Independent Safety Committee:***

Prof. Val Gebski; (University of Sydney/NHMRC Clinical Trial Centre)

Dr. Thomas Kimber; (Royal Adelaide Hospital/University of Adelaide) and

Prof. Alan Barber; (University of Auckland/Auckland City Hospital).

***Medical Monitor***

Dr Paul Wraight, Royal Melbourne Hospital

***Others***

Dr Sandeep Sampangi, Dr Rashida Ali, (Monash University) biobanking

Prof David Miller, University College London, for setting up MRI analysis facilities.

Prof. Lauren Krupp for free use of Fatigue Severity Scale

Prof. Leonid Churilov for Randomisation Schedules

Michael Ching and his staff at Austin Health Clinical Trials Pharmacy for Central IMP Depot services

***Investigators***

Prof Susanne Hodkinson, Liverpool Hospital

A/Prof Ernie Butler, Monash Medical Centre

A/Prof Cameron Shaw, Geelong Hospital

A/Prof Claire Fraser, Save Sight Institute, Sydney

Dr John Mottershead, Dunedin Hospital

## Analysis review

The statistical analysis plan was developed by KD, ALP, HB, RL, MS, and BVT and the primary analysis was conducted by KD, an independent review of the analysis was conducted by CZ confirming the primary analysis.
